# Surveillance of First-Generation H1-Antihistamine Use for Older Patients with Dementia in Japan: A Retrospective Cohort Study

**DOI:** 10.1155/2018/3406210

**Published:** 2018-07-02

**Authors:** Toshiki Maeda, Akira Babazono, Takumi Nishi

**Affiliations:** ^1^Department of Preventive Medicine and Public Health, Faculty of Medicine, Fukuoka University, 8-19-1 Nanakuma, Jonan-ku, Fukuoka 814-0180, Japan; ^2^Department of Healthcare Administration and Management, Graduate School of Healthcare Sciences, Kyushu University, 3-1-1 Maidashi, Higashi-ku, Fukuoka 812-8582, Japan

## Abstract

**Background:**

This study aimed to investigate the rate of first-generation H1-antihistamines use for older adults with dementia in Japan.

**Methods:**

The study design was retrospective cohort using claims data between fiscal years 2010 and 2013. Subjects were 75 years or older, diagnosed with dementia, and given H1-antihistamines orally during the study period after being diagnosed with dementia. We investigated the cumulative number of oral H1-antihistamines administered and the relationship between first-generation H1-antihistamine use and each explanatory variable using crude and adjusted odds ratio.

**Results:**

The cumulative total for use of first-generation H1-antihistamine for older adults with dementia accounted for 32.1% of all antihistamine medication. The majority of first-generation H1-antihistamine prescriptions were indicated for cold treatment. Those with upper respiratory infection or asthma had a significantly positive relationship with first-generation H1-antihistamine use.

**Conclusion:**

The study showed that first-generation H1-antihistamine drugs were highly prescribed in older adults with dementia in Japan.

## 1. Introduction

In Japan, there has been an unprecedented growth of the aging society. The number of people aged 65 years or older was 33.8 million in 2015, accounting for more than one-quarter of the total population [1]. Dementia is one of the major burdens related to aging [2]. It is estimated that the number of older adults aged 65 years or older suffering from dementia is approximately 4.62 million, indicating that one out of seven Japanese older adults has dementia [3]. Thus, there is an urgent need to establish effective care policies and provide high quality care for older Japanese adults, particularly those suffering from cognitive impairment.

Older adults tend to have adverse effects induced by medication because of lower metabolism and clearance inherent in the aging process [4]. Therefore, there are several medications that may induce adverse effects on older adults [5]. H1-antihistamines are a frequently used medication in routine clinical practice, and these are among the medications that should be avoided when treating older adults. H1-antihistamine drugs are classified into first- or second-generation antihistamines [6]. First-generation H1-antihistamines have anticholinergic effects [5], which may lead to constipation and dry mouth [6]. These agents also pass the blood brain barrier easily and therefore cause drowsiness, sedation, somnolence, and fatigue [7]. Thus, first-generation H1-antihistamines are also referred to as sedative H1-antihistamines [7]. Older adults, in particular, those with dementia, are vulnerable to first-generation H1-antihistamine medication not only because of its sedative effects, but also because these drugs may lead to agitation and delirium [6, 8]. Thus, frequent use of first-generation H1-antihistamines is potentially" inappropriate, in particular for vulnerable older adults.

Reportedly, first-generation H1-antihistamines are frequently used for older Japanese adults, although the data are not shown [9]. Few studies have investigated the first-generation H1-antihistamine use. Additionally, few studies focus on patterns of antihistamine use among older adults with dementia in Japan. Therefore, this study aimed to investigate the rate of first-generation H1-antihistamine use and the cumulative H1-antihistamine use and to elucidate the factors related to first-generation H1-antihistamines for older adults with dementia in Japan. 

## 2. Subjects and Methods

This study was a retrospective cohort design that closely followed the internationally recognized Strengthening the Reporting of Observational Studies in Epidemiology guidelines [10]. We retrospectively analyzed claims data submitted to the Fukuoka Late Elders' Health Insurance between the fiscal year (Fy) 2010 and Fy 2013. In Japan, adults aged ≥75 years or those aged between 65 and 74 years with a specific disability are eligible for the Late Elders' Health Insurance. We assumed that adults aged between 65 and 74 years had a specific intractable disease; thus, we assigned those 75 years or older as our study subjects. We identified 67000 those diagnosed before H1-antihistamine use with dementia according to International Disease Classification 10th revision (ICD-10). Subjects with ICD10 codes 'F00', 'F01', 'F02', or 'F03' were defined as subjects with dementia. Then we observed the cumulative number of oral H1-antihistamine drugs administered during the study period. Oral H1-antihistamines were classified into first or second generation. We used the standardized pharmaceutical classification which was issued by Ministry of Health, Labour and Welfare for information of medical fee in Japan [11] and publication[12] to identify H1-antihistamines prescribed (Supplementary Table 1).

We assigned multi-ingredient cold medications into the first-generation H1-antihistamine category as these contained first-generation H1-antihistamine.

Regarding disease, we identified those that had allergic rhinitis (ICD10; J30), asthma (ICD10; J45), eczema (ICD10; L30), urticaria (ICD10; L50), pruritus (ICD10; L29), or upper respiratory infection (URI) (ICD10; J00-06). We excluded promethazine hydrochloride, which was classified as a first-generation antihistamine because this drug is indicated for Parkinson's disease in Japan and our aim was surveillance of potentially inappropriate medication which could change to less adverse alternatives in those with URI or other allergic diseases. We also excluded dimenhydrinate because it is indicated for the treatment of vertigo. This study was approved by the Institutional Review Board of Kyushu University (Clinical Bioethics Committee of the Graduate School of Healthcare Sciences, Kyushu University).

### 2.1. Definition of Variables

The explanatory variables were sex, age, antidementia medication use, Fy of the H1-antihistamine use, comorbidities, the number of beds, and location of care facilities.

Age was classified into 75 to 79 years, 80 to 84 years, 85 to 89 years, and 90 or older. Comorbidities were classified according to Charlson comorbidity index (CCI) [13]. The number of beds was stratified into 0 to 19, 20 to 199, and 200 or more. We identified the secondary tier of medical care (STM) in Fukuoka prefecture that each facility belonged to. STM is a unit of secondary care governed by a corresponding prefecture according to Japan's Medical Service Law [14].

### 2.2. Statistical Analyses

We evaluated the relationships between first-generation H1-antihistamine use and each explanatory variable using percentage, crude odds ratio (OR), and adjusted odds ratio (AOR). Then, we repeated the same analysis after excluding those who received cold medicine. We employed logistic regression analysis with first-generation H1-antihistamine use as the outcome variable and sex, age, disease, comorbidities, antidementia medication use, and the number and location of care facilities as explanatory variables for all subjects. Then, the same analysis was conducted after excluding subjects receiving cold medicine. We analyzed subjects as clusters [15] because the study assessed the cumulative number of subjects and some subjects were observed repeatedly. As to STM, we set the least frequent STM as a reference.

We used STATA version 14 (StataCorp, College Station, TX, USA) for statistical analysis. All reported p-values were two-tailed, and the level of significance was set at p<0.05.

## 3. Results

### 3.1. Descriptive Data

The total number of subjects was 12,658 and the number of subjects treated with first-generation of H1-antihistamine was 8272 (65.3%). The number of those given cold medicine at least once was 7227, which accounted for 57.1% of all patients. As for cumulative total of medication use, first-generation H1-antihistamine accounted for 32.1%. The percentage of cold medication was 22.7% of the cumulative H1-antihistamine use, which accounted for 70.6% of first-generation H1-antihistamines. Minimum and maximum frequencies of first-generation H1-antihistamine and cold medication were 1 and 140 and 1 and 90, respectively.

### 3.2. The Relationships between First-Generation H1-Antihistamine and Explanatory Variables

**Figure 1:**
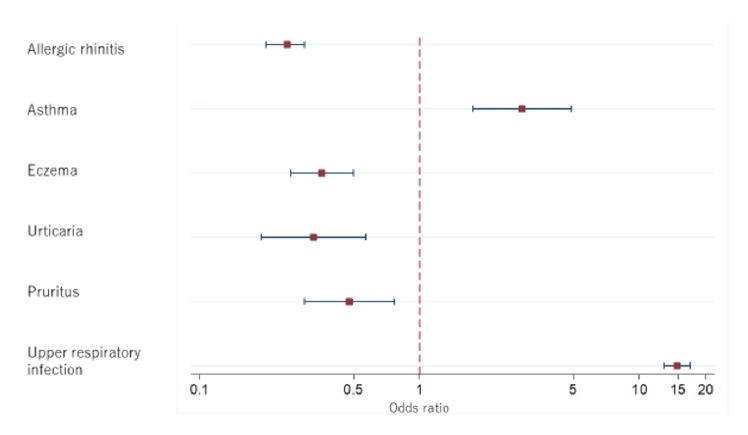
Odds ratios of first-generation H1-antihistamine use among diseases.

The rate, OR, and AOR of first-generation H1-antihistamine use are shown in Figure 1 and Supplementary Table 2. The subjects aged 90 years or older tended to be used first-generation H1-antihistamine use fewer than reference category after controlling for confounding variables (AOR 0.73 [0.59–0.91] p=0.004). Female patients were positively associated with first-generation H1-antihistamines use (AOR 1.43 [1.23–1.65]) p<0.001). Those with asthma and URI were given first-generation H1-antihistamine significantly more frequently than those without asthma and URI. The AOR for subjects with asthma and URI was approximately 3-fold and more than 10-fold higher than those without asthma and URI, respectively (asthma: AOR 2.93 [1.75–4.91], p<0.001; URI: AOR 14.84 [12.98–16.97]), p<0.001). Subjects with allergic rhinitis, eczema, urticaria, and pruritus were significantly and negatively related to first-generation H1-antihistamine use than those without allergic rhinitis, eczema, urticaria and pruritus (allergic rhinitis AOR 0.25 [0.20–0.30], p<0.001; eczema AOR 0.36 [0.26–0.50], p<0.001; urticaria AOR 0.33 [0.19–0.57], p<0.001; pruritus AOR 0.48 [0.30–0.77], p=0.002). Subjects receiving antidementia medication had approximately 20% lower odds of first-generation H1-antihistamine use and the difference was statistically significant (AOR 0.78 [0.68–0.90] p=0.001). As for comorbidities, subjects with congestive heart failure were negatively associated with first-generation H1-antihistamine use (congestive heart failure AOR 0.73 [0.59–0.90] p=0.004). Care facilities having 20 to 199 beds and those with 200 or more beds used first-generation H1-antihistamine significantly more frequently (beds 20–199: AOR 1.40 (1.17–1.68) p<0.001, 200 or more: AOR 1.55 (1.30–1.85) p<0.001) than that of other facilities. With regard to Fy, the prescription rate tended to be lower as time elapsed. The AORs of first-generation H1-antihistamine use were significantly lower in Fy 2012 and Fy 2013 than in Fy 2010 (Fy 2012: AOR 0.87 [0.77–1.00], p=0.045; Fy 2013: AOR 0.78 [0.68–0.89] p<0.001). Care facilities located in STM 2, 4, 7, 9, 12, and 13 had significant positive relationships with H1-antihistamine use compared with STM8. The odds ratio at maximum STM was approximately more than 2-fold that of minimum STM

### 3.3. The Relationships between First-Generation H1-Antihistamine and Explanatory Variables, Except for Cold Medication

**Figure 2:**
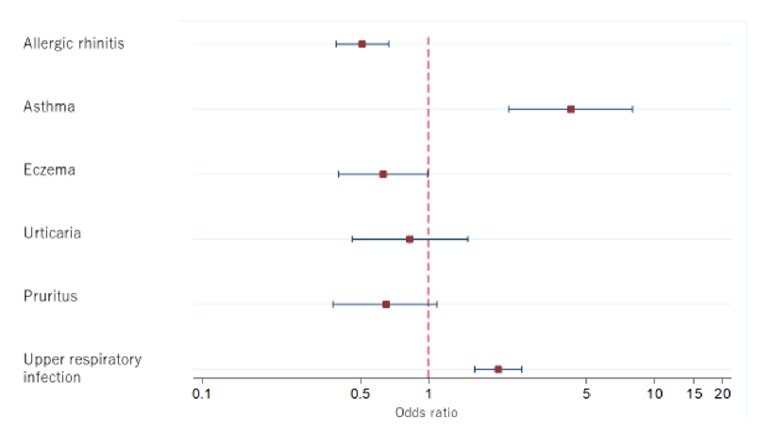
Odds ratios of first-generation H1-antihistamine use excluding cold medication among diseases.

The rate and OR and AOR of first-generation H1-antihistamine use after excluding cold medication are shown in Figure 2 and Supplementary Table 3. Those with asthma and URI had a significant positive relationship with H1-antihistamine use, even after excluding cold medication (asthma: AOR 4.27 [2.27–8.03], p<0.001; URI AOR 2.04 [1.60–2.60], p<0.001). Subjects with allergic rhinitis or eczema had a significant and negative association with first-generation H1-antihistamine use (allergic rhinitis: AOR 0.51 [0.39–0.67], p<0.001; eczema: AOR 0.63 [0.40–0.99], p=0.045). The AOR of first-generation H1-antihistamine use was significantly lower among subjects receiving antidementia medications than among those without antidementia treatment (AOR 0.75 [0.58–0.96] p=0.021). Subjects with mild liver disease were given first-generation H1-antihistamine with significantly higher odds (AOR 3.62 [1.56–8.38] p=0.003).

After excluding for cold medicine, care facilities with 20–199 beds had a significantly higher odds ratio of first-generation H1-antihistamine use. The odds ratio of first-generation H1-antihistamine use was significantly higher in Fy 2013 (AOR 0.76 [0.59–0.98] p=0.034). Care facilities located in STM 7, 2, 4, 9, or 13 had significantly higher odds of first-generation H1-antihistamine use than those in STM 1.

## 4. Discussion

The present study investigated first-generation H1-antihistamine use for older adults with dementia. Those with URI and asthma had a significantly positive relationship with first-generation H1-antihistamine use. In contrast, other allergic diseases were not associated with an increased use of first-generation H1-antihistamine drugs. We considered the reason for the above is that the pathophysiology of allergic diseases, like allergic rhinitis and eczema, are relatively well known. Histamine plays an essential role in the pathophysiology of these diseases [16–18]. Thus, second-generation H1-antihistamine was used more often in such cases because these agents have similar efficacy to first-generation H1-antihistamines, but do not exert adverse effects [19, 20] such as drowsiness, sedation, somnolence, and fatigue [7]. Conversely, a common cold is defined as a self-limited illness confined to the upper respiratory tract mainly caused by viral infection [21]. The clinical presentation of a cold includes sore throat, nasal discharge, nasal congestion, and sneezing [21]. The pathophysiology of common colds, however, is not completely understood, unlike allergic rhinitis or eczema, and various cytokines might be associated with the occurrence of cold rather than solely histamines [21]. Although various medications are used to treat colds, it is reported that first-generation H1-antihistamines are relatively more effective than second-generation antihistamines [21, 22]. This may be attributed mainly to the anticholinergic effects rather than antihistamine effects [21]. However, it has also been reported that the effect of first-generation H1-antihistamines was limited and associated with more side effects including drowsiness, sedation, somnolence, fatigue, and weight gain [7, 22]. Moreover recent studies reported that anticholinergics including first-generation antihistamine drugs increase the incidence of dementia [23, 24]. Hence, first-generation H1-antihistamine drugs should not be used, in particular, for older people with dementia. Nevertheless, the present study revealed that first-generation H1-antihistamine drugs were frequently used for those with URI. This might be because physicians aim to relieve patient symptoms derived from patient preference or medication indications that are approved by healthcare authorities. However, as there were small area variations after controlling for patients characteristic, we could not rule out the possibility that physician practice style [25] affected variations of first-generation H1-antihistamine use. This usage was considered unwarranted [26] because treatment with first-generation H1-antihistamine can lead to adverse effects in vulnerable aging adults with dementia. In this particular case, the physician should not follow patient preferences, but they should rather balance the efficacy and adverse effects of first-generation H1-antihistamine drugs in this vulnerable population. Interestingly, subjects with asthma were prescribed first-generation H1-antihistamine more frequently. Although the effects of H1-antihistamine agents may benefit those having asthma with allergic predisposition, the use of second-generation H1-antihistamines is recommended [27, 28] because first-generation agents can cause adverse effects such as excessive drying of the mucus membranes [29] in addition to the sedation effects. Furthermore, only second-generation antihistamines are approved for asthma treatment in Japan. Nevertheless, in this study we found that first-generation antihistamine was used more often for subjects with asthma. We cannot elucidate the reason of this frequent usage, which seems inappropriate, but further investigations are needed clarify this choice of treatment.

We also found that those with mild liver disease had significantly higher odds for first-generation H1-antihistamines use. This could be because mild liver diseases corresponding to Charlsons' comorbidity index included chronic hepatitis and primary biliary cholangitis that caused pruritus hence first-generation H1-antihistamines might be used for treating pruritus [30, 31].

The strength of this study is that the claims data used in this study encompassed almost all older adults residing in Fukuoka prefecture and captured nearly all medications given during that period. This study also had some limitations. Our dataset did not include elderly subjects without dementia so that we could not compare the prescription of sedating antihistamines in elderly subjects with and without dementia. We were not able to observe whether the patients actually took the drugs prescribed. Further, we did not evaluate over-the-counter drugs. Patient activities of daily life and clinical data were not available either. The claim data we used did not have residential information including long term care facility; hence we could not control the residential status. The present study did not focus on care contents and outcome but on processes and variability. Therefore, further studies are needed to examine appropriateness of care contents for older people with dementia by linking claim data with chart or other administrative data.

## 5. Conclusion

This study investigated the current clinical status on first-generation H1-antihistamine use in older Japanese adults with dementia. The cumulative use of first-generation H1-antihistamines accounted for 32.1% and cold medication comprised the great majority of the medication given. Older subjects with dementia with URI and asthma had a significantly positive relationship with first-generation H1-antihistamine use. Our study findings suggest that there could be unwarranted use of first-generation H1-antihistamine in the management of older adults with dementia. Further studies are needed to examine the appropriateness of care for older adults with dementia.

## 6. Impact Statement

The study showed that there could be unwarranted use of first-generation H1-antihistamine in the management of older adults with dementia. Continuous surveillance would be needed for prescription of potentially harmful medication.

## Data Availability

The data used to support the findings of this study are available from the corresponding author after ethical committee's permission.

## Abbreviations

Fy: Fiscal year
ICD-10: International Disease Classification 10th revision
CCI: Charlson comorbidity index
URI: Upper respiratory infection
STM: Secondary tier of medical care
AOR: Adjusted odds ratio.

## Supplementary Materials

Supplementary Materials Supplementary Table 1: list of first-generation H1-antihistamine drugs used for analyses. Supplementary Table 2: rate, crude, and adjusted odds ratio of first-generation H1-antihistamine use. Supplementary Table 3: rate, crude, and adjusted odds ratio of first-generation H1-antihistamine use after excluding cold medication.
