# Supplementary material for: Surveillance of First-Generation H1-Antihistamine Use for Older Patients with Dementia in Japan: A Retrospective Cohort Study
Source: Curr Gerontol Geriatr Res. 2018 Jul 2;2018:3406210. doi: 10.1155/2018/3406210 (PMC6051324; doi:10.1155/2018/3406210)
Supplement: Supplementary Materials — Supplementary Table 1: list of first-generation H1-antihistamine drugs used for analyses. Supplementary Table 2: rate, crude, and adjusted odds ratio of first-generation H1-antihistamine use. Supplementary Table 3: rate, crude, and adjusted odds ratio of first-generation H1-antihistamine use after excluding cold medication. [file 3406210.f1.pdf]

Supplementary Table1. List of first-generation H1-antihistamine drugs used for analyses

Diphenhydramine hydrochloride
Clemastine fumarate
dl-chrorpheniraine maleate
d-chrorpheniraine maleate
Triprolidine hydrochloride hydrate
Alimemazine tartrate
Hydroxyzine
Homochlorcyclizine hydrochloride
Cyproheptadine hydrochloride

Supplementary Table2. Rate, crude, and adjusted odds ratio of first-generation H1-antihistamine use

	N	%	OR	95%CI		p	AOR	95%CI		p
Gender										
Male	6332	27.4	Reference				Reference			
Female	18417	34.2	1.38	1.33	1.42	<0.001	1.43	1.23	1.65	<0.001
Age										
75-79	4962	34.6	Reference							
80-84	8100	33.2	0.94	0.90	0.98	0.004	0.91	0.78	1.07	0.251
85-89	7244	31.8	0.88	0.84	0.92	<0.001	0.84	0.70	1.01	0.061
90-	4443	28.7	0.76	0.72	0.80	<0.001	0.73	0.59	0.91	0.004
Disease										
Allergic rhinitis	450	21.4	0.57	0.51	0.63	<0.001	0.25	0.20	0.30	<0.001
Asthma	153	57.1	2.82	2.21	3.59	<0.001	2.93	1.75	4.91	<0.001
Eczema	97	14.2	0.35	0.28	0.43	<0.001	0.36	0.26	0.50	<0.001
Urticaria	31	13.4	0.33	0.22	0.48	<0.001	0.33	0.19	0.57	<0.001
Pruritus	65	14.3	0.35	0.27	0.46	<0.001	0.48	0.30	0.77	0.002
URI	6365	80.0	11.02	10.41	11.68	<0.001	14.84	12.98	16.97	<0.001
Medication										
Antidementia	10422	30.0	0.84	0.81	0.86	<0.001	0.78	0.68	0.90	0.001
Comorbidities										
Acute myocardial infarction	250	33.7	1.08	0.92	1.25	0.350	1.03	0.64	1.67	0.903
Cerebrovascular accident	7353	31.9	0.98	0.95	1.02	0.337	1.03	0.88	1.20	0.712
Collagen disease	282	33.0	1.04	0.90	1.20	0.597	1.01	0.50	2.05	0.980
Congestive heart failure	2752	27.1	0.76	0.72	0.80	<0.001	0.73	0.59	0.90	0.004
Diabetes no complication	56	33.5	1.07	0.77	1.47	0.700	0.91	0.41	2.02	0.820
Diabetes with complication	165	32.8	1.03	0.86	1.24	0.750	1.07	0.61	1.90	0.810
Hemipalysis	53	33.3	1.06	0.76	1.47	0.747	1.17	0.47	2.96	0.733
Metastatic malignancy	53	41.7	1.51	1.06	2.15	0.021	1.28	0.53	3.10	0.578
Mild liver disease	654	40.3	1.44	1.30	1.59	<0.001	1.87	0.97	3.60	0.063
Moderate to severe liver disease	21	24.1	0.67	0.41	1.10	0.112	0.39	0.12	1.26	0.117
Peptic ulcer	1787	32.6	1.02	0.96	1.08	0.448	0.95	0.70	1.29	0.762
Renal disease	494	27.1	0.78	0.70	0.87	<0.001	0.84	0.57	1.24	0.374
Peripheral vascular disease	342	25.6	0.72	0.64	0.82	<0.001	0.63	0.36	1.10	0.103
Primary neoplasm	695	27.1	0.78	0.71	0.85	<0.001	0.78	0.54	1.12	0.174
Pulmonary disease	3492	29.8	0.88	0.84	0.92	<0.001	0.83	0.68	1.02	0.076
Number of bed										
0-19	13428	29.6	Reference				Reference			
20-199	5025	34.3	1.24	1.19	1.29	<0.001	1.40	1.17	1.68	<0.001
200-	6296	37.2	1.41	1.36	1.46	<0.001	1.55	1.30	1.85	<0.001
Fiscal year										
2010	4840	36.0	Reference				Reference			
2011	6069	34.8	0.95	0.91	0.99	0.029	0.97	0.87	1.08	0.605
2012	6774	31.6	0.82	0.79	0.86	<0.001	0.87	0.77	1.00	0.045
2013	7066	28.7	0.71	0.68	0.75	<0.001	0.78	0.68	0.89	<0.001
STM										
1	5908	28.9	1.40	1.30	1.51	<0.001	1.37	0.98	1.93	0.069
2	1026	37.3	2.06	1.86	2.28	<0.001	1.97	1.26	3.08	0.003
3	444	32.8	1.68	1.48	1.92	<0.001	1.47	0.82	2.65	0.199
4	1466	34.0	1.78	1.62	1.95	<0.001	1.82	1.21	2.76	0.004
5	473	30.3	1.50	1.33	1.71	<0.001	1.29	0.68	2.43	0.438
6	1518	29.0	1.41	1.29	1.54	<0.001	1.24	0.84	1.82	0.273
7	1168	36.5	1.99	1.80	2.19	<0.001	2.15	1.31	3.53	0.002
8	1129	22.5	Reference				Reference			
9	1380	38.9	2.20	2.00	2.42	<0.001	1.75	1.12	2.73	0.015
10	501	31.2	1.57	1.38	1.78	<0.001	1.61	0.97	2.67	0.067
11	1181	37.3	2.06	1.87	2.27	<0.001	1.36	0.89	2.09	0.159
12	6938	34.1	1.79	1.67	1.92	<0.001	1.69	1.21	2.37	0.002
13	1617	36.2	1.96	1.79	2.14	<0.001	1.71	1.02	2.85	0.040
Total	24749	32.1								

URI: Upper respiratory infection, STM: secondary tier of medical care

Supplementary Table3. Rate, crude and adjusted odds ratio of first-generation H1-antihistamine use after excluding cold medication

	N	%	OR	95%CI		p	AOR	95%CI		p
Gender										
Male	1988	10.6		Reference				Reference		
Female	5277	13.0	1.26	1.19	1.33	<0.001	1.26	0.96	1.65	0.090
Age										
75-79	1319	12.3		Reference				Reference		
80-84	2090	11.4	0.91	0.85	0.98	0.012	0.91	0.67	1.22	0.516
85-89	2241	12.6	1.02	0.95	1.10	0.511	1.01	0.72	1.41	0.956
90-	1615	12.8	1.04	0.96	1.12	0.326	1.02	0.70	1.49	0.898
Disease										
Allergic rhinitis	131	7.3	0.56	0.47	0.67	<0.001	0.51	0.39	0.67	<0.001
Asthma	64	35.8	4.03	2.97	5.48	<0.001	4.27	2.27	8.03	<0.001
Eczema	47	7.4	0.58	0.43	0.78	<0.001	0.63	0.40	0.99	0.045
Urticaria	21	9.5	0.75	0.48	1.18	0.220	0.83	0.46	1.50	0.544
Pruritus	31	7.4	0.57	0.40	0.83	0.003	0.65	0.38	1.09	0.105
URI	363	18.6	1.67	1.49	1.88	<0.001	2.04	1.60	2.60	<0.001
Medication										
Antidementia	2765	10.2	0.71	0.67	0.74	<0.001	0.75	0.58	0.96	0.021
Comorbidities										
Acute myocardial infarction	42	7.9	0.61	0.45	0.84	0.002	0.65	0.31	1.36	0.253
Cerebrovascular accident	904	10.9	1.06	1.01	1.12	0.030	1.10	0.84	1.46	0.487
Collagen disease	129	18.4	1.63	1.34	1.98	<0.001	1.42	0.47	4.25	0.530
Congestive heart failure	2274	12.7	0.86	0.80	0.93	<0.001	0.80	0.55	1.17	0.251
Diabetes no complication	35	9.4	0.52	0.25	1.06	0.072	0.54	0.10	3.01	0.483
Diabetes with complication	6	5.4	0.74	0.52	1.05	0.096	0.92	0.38	2.21	0.845
Hemipalysis	9	10.8	0.41	0.18	0.93	0.032	0.37	0.04	3.48	0.383
Metastatic malignancy	8	6.7	0.87	0.44	1.75	0.704	0.78	0.10	6.28	0.818
Mild liver disease	345	26.3	2.64	2.33	2.99	<0.001	3.62	1.56	8.38	0.003
Moderate to severe liver disease	0	0.0		－						
Peptic ulcer	456	11.0	0.88	0.80	0.97	0.013	0.81	0.46	1.45	0.478
Renal disease	217	10.4	0.91	0.78	1.07	0.272	1.00	0.49	2.05	0.998
Peripheral vascular disease	952	10.4	0.59	0.47	0.74	<0.001	0.51	0.20	1.33	0.171
Primary neoplasm	82	7.6	0.83	0.72	0.96	0.010	0.93	0.46	1.88	0.833
Pulmonary disease	169	11.3	0.81	0.75	0.87	<0.001	0.81	0.56	1.17	0.266
Number of bed										
0-19	3706	10.4		Reference				Reference		
20-199	1823	15.9	1.63	1.54	1.74	<0.001	1.62	1.20	2.20	0.002
200-	1736	14.0	1.41	1.33	1.50	<0.001	1.23	0.87	1.75	0.244
Fiscal year										
2010	1433	14.3		Reference				Reference		
2011	1785	13.6	0.94	0.87	1.02	0.123	0.96	0.79	1.16	0.665
2012	2006	12.0	0.82	0.76	0.88	<0.001	0.87	0.68	1.11	0.259
2013	2041	10.4	0.70	0.65	0.75	<0.001	0.76	0.59	0.98	0.034
STM										
1	1336	8.4		Reference				Reference		
2	390	18.5	2.46	2.18	2.79	<0.001	2.67	1.50	4.74	0.001
3	132	12.7	1.58	1.30	1.91	<0.001	1.53	0.56	4.16	0.406
4	423	12.9	1.62	1.44	1.81	<0.001	1.80	1.01	3.22	0.047
5	169	13.5	1.69	1.43	2.01	<0.001	1.56	0.72	3.38	0.260
6	520	12.3	1.52	1.37	1.69	<0.001	1.50	0.92	2.42	0.100
7	541	21.1	2.90	2.60	3.24	<0.001	3.54	1.94	6.46	<0.001
8	464	10.6	1.30	1.16	1.45	<0.001	1.17	0.69	1.99	0.562
9	396	15.5	1.99	1.76	2.25	<0.001	2.03	1.13	3.67	0.019
10	197	15.2	1.94	1.65	2.29	<0.001	1.77	0.91	3.42	0.092
11	296	13.0	1.62	1.42	1.86	<0.001	1.49	0.80	2.80	0.212
12	1789	11.8	1.46	1.35	1.57	<0.001	1.44	1.00	2.10	0.053
13	612	17.7	2.34	2.11	2.59	<0.001	2.13	1.06	4.29	0.035
Total	7265	12.2								

URI: Upper respiratory infection, STM: Secondary tier of medical care
